# A Bayesian network approach to study host and viral genetic correlates of HIV-1 disease progression

**DOI:** 10.1186/1742-4690-8-S2-P70

**Published:** 2011-10-03

**Authors:** Joke Snoek, Concepción Casado, Sara Colombo, Andri Rauch, Soledad García, Raquel Martinez, Kristof Theys, Ricardo Khouri, Huldrych F Günthard, Amalio Telenti, Cecilio López-Galíndez, Anne-Mieke Vandamme

**Affiliations:** 1 Clinical and Epidemiological Virology, Rega Institute, Katholieke Universiteit Leuven, B-3000 Leuven, Belgium; 2 Institute of Microbiology, University Hospital Center, University of Lausanne, Lausanne, Switzerland; 3 Centro Nacional de Microbiología (CNM), Instituto de Salud Carlos III, Majadahonda, 28220 Madrid, Spain; 4 University Clinic of Infectious Diseases, University Hospital Bern and University of Bern, Bern, Switzerland; 5 Centro Sanitario Sandoval (CSS), IMSALUD, Comunidad Autónoma de Madrid, 28010 Madrid, Spain; 6 Division of Infectious Diseasesand Hospital Epidemiology, University of Zurich, Zurich, Switzerland

## Background

HIV disease progression is very variable among infected patients. Using classical statistical methods based on a selected number of markers, Casado et al [1] identified a number of host and viral genetic correlates for the clinical definitions of HIV-1 disease progression: elite controllers, long term non progressors including viremic controllers and clinical non progressors, regular progressors and rapid progressors.

## Materials and methods

Host genetic and viral data for 64 patients as described by Casado et al were transformed to Boolean variables and used in a Bayesian Network (BN) learning approach, using the online version of the B-Course software (http://b-course.cs.helsinki.fi/obc/), scoring models by maximizing the posterior probability of the model. The predictive value of the network for classifying the clinical definitions, was investigated using exact Bayesian inference in the network The network arcs that were weighing most in the predictive power of the network were uncovered by evaluating for each arc how predictive the network still was when omitting this arc.

## Results

To visualize the various dependencies among clinical definitions, and host and viral factors, we used a Bayesian network representation of the data. A number of variables that were redundant (being part of the definition of the groups) or non-discriminative were left out for inferring the network: viral load, proviral load, predicted X4/R5 phenotype and nature and charge, ethnicity and age. The network correctly captured many known correlations in the data, for example, patterns of linkage disequilibrium among genetic markers (i.e., *HLA-A* and *ZNRD1*, *HLA-B* and *HLA-C* alleles, *CCR5* haplotypes), and provided numerical support to the various dependencies. Clinical definitions (class) were directly dependent on viral evolution expressed as "viral dating", protective *HLA-B* alleles, and *HLA-C* genotypes. One variable, *CCR2 V64l rs1799864*, was not retained in the network. The results were consistent with what Casado et al described in their paper.

## Conclusions

We applied a Bayesian approach to the joint analysis of the diverse host and viral data. The resulting network provided a comprehensive and hierarchical structure of the various dependencies; it identified viral evolution and *HLA-B* and *HLA-C* alleles as key correlates of clinical definitions of disease progression, confirming the results obtained by classical statistics. We feel that the Bayesian approach is well suited to quickly explore large datasets, saving time by prioritizing subsequent statistical confirmation of the associations found. Such Bayesian networks could also be applied for predicting the clinical course of the individual.
